# Heterogeneous Circulating Tumor Cells in Sarcoma: Implication for Clinical Practice

**DOI:** 10.3390/cancers13092189

**Published:** 2021-05-02

**Authors:** Chiara Agnoletto, Chiara Caruso, Cecilia Garofalo

**Affiliations:** Advanced Translational Research Laboratory, Veneto Institute of Oncology IOV—IRCCS, 35127 Padua, Italy; chiara.agnoletto@iov.veneto.it (C.A.); chiara.caruso@iov.veneto.it (C.C.)

**Keywords:** sarcoma, CTC, epithelial–mesenchymal plasticity, clinical practice

## Abstract

**Simple Summary:**

The present review is aimed to discuss the relevance of assaying for the presence and isolation of circulating tumor cells (CTCs) in patients with sarcoma. Just a few studies have been performed to detect and enumerate viable CTCs in sarcoma and a majority of them still represent proof-of-concept studies, while more frequently tumor cells have been detected in the circulation by using the PCR-based method. Nevertheless, recent advances in technologies allowed detection of epithelial–mesenchymal transitioned CTCs from patients with mesenchymal malignancies, despite results being mostly preliminary. The possibility to identify CTCs holds a great promise for both applications of liquid biopsy in sarcoma for precision medicine, and for research purposes to pinpoint the mechanism of the metastatic process through the characterization of tumor mesenchymal cells. Coherently, clinical trials in sarcoma have been designed accordingly to detect CTCs, for diagnosis, identification of novel therapeutic targets and resistance mechanisms of systemic therapies, and patient stratification.

**Abstract:**

Bone and soft tissue sarcomas (STSs) represent a group of heterogeneous rare malignant tumors of mesenchymal origin, with a poor prognosis. Due to their low incidence, only a few studies have been reported addressing circulating tumor cells (CTCs) in sarcoma, despite the well-documented relevance for applications of liquid biopsy in precision medicine. In the present review, the most recent data relative to the detection and isolation of viable and intact CTCs in these tumors will be reviewed, and the heterogeneity in CTCs will be discussed. The relevance of epithelial–mesenchymal plasticity and stemness in defining the phenotypic and functional properties of these rare cells in sarcoma will be highlighted. Of note, the existence of dynamic epithelial–mesenchymal transition (EMT)-related processes in sarcoma tumors has only recently been related to their clinical aggressiveness. Also, the presence of epithelial cell adhesion molecule (EpCAM)-positive CTC in sarcoma has been weakly correlated with poor outcome and disease progression, thus proving the existence of both epithelial and mesenchymal CTC in sarcoma. The advancement in technologies for capturing and enumerating all diverse CTCs phenotype originating from these mesenchymal tumors are presented, and results provide a promising basis for clinical application of CTC detection in sarcoma.

## 1. Introduction

Sarcomas are an uncommon and heterogeneous group of mesenchymal malignant tumors originating from bone, cartilage as well as other mesenchymal tissues, such as muscle, fat, peripheral nerves, fibrous, or related tissues [1]. The most common soft-tissue histologic subtypes are high-grade undifferentiated pleomorphic sarcoma, gastrointestinal stromal tumors (GIST), liposarcoma, and leiomyosarcoma, while bone sarcomas include osteosarcoma, chondrosarcoma, and Ewing sarcoma [2,3,4]. Although the raised progress in discovering genetic aberrations and their functions in these tumors, the major therapeutic option for the majority of local recurrence and metastatic sarcomas remain cytotoxic chemotherapy. Chemotherapy remains controversial in the adjuvant curative setting for the most common types of adult soft tissue sarcomas (STSs) and recent improvements in bone sarcomas have been achieved only thanks to the use of intensive chemotherapy, thus paying the price of short and long-term side effects. Therefore, conventional treatments remain the standard and sometimes the sole option for patients with sarcomas.

Additionally, metastatic disease is the most crucial factor that determines the survival of the great majority of newly diagnosed patients with sarcoma [5]. Even if the incidence is low, sarcoma presents a high mortality rate due to high metastatic potential, late diagnosis, and relapse [6]. Thus, new tools need to be developed to help in the identification of patients’ responses or resistances to specific therapies and the prediction of toxicity side effects due to therapies. In this contest, recent studies on pharmacogenomics biomarkers described their modulation due to both conventional and new chemotherapeutics drugs, in several sarcoma histotypes [7]. Radiographic imaging is routinely performed for follow-up after surgery, thus depicting the effects of neoadjuvant therapies [8], but often cannot detect metastatic loci and does not differentiate tumor histology and not accurately predict the survival of high-risk patients [9], as recently discussed in STS [10]. Conventional therapy combines neoadjuvant chemotherapy followed by surgical removal, and postoperative chemotherapy [11,12,13]. In patients with localized disease long-term survival has dramatically improved through neoadjuvant and adjuvant chemotherapy, supporting the presence of micrometastatic disease at diagnosis in most patients [14,15]. However, ~50% of relapsing of patients with sarcomapresent chemo-sensitive tumors [16], and patients with overt metastases still have a poor prognosis. Despite multidisciplinary therapies, metastasis remains a critical issue [17]. Moreover, the response rates toward novel molecular-targeting drugs are not very high [18,19], and new treatment strategies are required [20]. Therefore, the presence of occult micro-metastases should be assessed at diagnosis, during therapy, and follow-up and their impact should be confirmed on progression and outcome. A potential new approach for the early detection of relapse is to capture CTCs from peripheral blood of patients with sarcoma who are in remission. The availability of a non-invasive method allowing sensitive detection, reliable identification, and molecular characterization of CTC potentially has great clinical value to help individualized therapy, for initial staging of the disease, for early detection of relapse, to improve prognosis and predict response to chemotherapy, and for identification of non-responders to therapeutic interventions. Recently, several circulating biomarkers have been proven to be useful in order to predict metastasis and to assess tumor activity, including CTCs, cell-free DNA (cfDNA), and microRNAs [21,22,23], confirming the diagnosis of standard imaging, histological techniques, and having prognostic value.

In the review, we discussed the most recent data relative to the detection and isolation of viable and intact CTCs in these tumors, and the heterogeneity in CTCs. Conversely, more recent literature extensively reported data discussing the relevance and implications of detecting extracellular exosomes and nucleic acids (both circulating tumor DNA, ctDNAs, and cfRNAs) in sarcoma [24,25,26,27].

In this contest, a method that detects CTCs and circulating cancer stem cells [28,29] would be most useful in assessing invasiveness, accurate prognosis, drug susceptibility, and resistance to therapy [30]. Briefly, the metastatic cascade consists of sequential steps, which include: (1) the intravasation of circulating tumor cells (CTCs), released from the primary tumor into the systemic circulation; (2) the extravasation of a small subset of neoplastic cells through the capillary endothelium of distant organs; and (3) the establishment of an even fewer number of cells and proliferation into clinically detectable secondary tumors in the new tissue environment [31,32,33,34,35,36]. Several technologies have been developed for CTCs capture and enumeration [37,38,39,40], and CTC counts have been proven to act as a predictive biomarker in solid tumors, correlating with poor outcomes, disease progression, and metastases [41,42,43,44,45]. The CellSearch™ System has been extensively validated in large patient cohorts for CTC enumeration, with the aim to identify patients at high-risk with decreased progression-free survival (PFS) and overall survival (OS) in breast, prostate, and colorectal cancers [46,47]. Also, several studies have proven the utility of molecular profiling of CTC for prognosis prediction, patient stratification, and therapeutic response monitoring [41,45].

## 2. CTC Heterogeneity: Epithelial–Mesenchymal Transitioned and Stem Cell-Like CTCs

The most recent improvements in microfluidic devices, together with new insight in single-cell-resolution next-generation sequencing (NGS) and mass cytometry technologies [48,49,50,51] have led to a better knowledge of the metastatic process. Thus, several studies identified the oligoclonal CTCs, the molecular profiling of CTCs, which differ compared to primary and metastatic tumors, and the identification of gene expression signatures in metastatic precursors and diverse CTC subsets [52]. Indeed, CTCs are heterogeneous at multiple levels and only a fraction of them consists of metastatic precursors [53,54,55,56], thus confirming the need to study CTCs at the single-cell level to pinpoint the mechanisms of metastasis.

The early steps of metastasis rely on the activation of the epithelial–mesenchymal transition (EMT) process of static epithelial tumor cells, into an acquired mesenchymal phenotype, with functional and dynamic changes in cell structure, migration, and invasion [32]. EMT is orchestrated by conserved inducing signals, transcriptional regulators, and effectors [32,57,58,59], in response to the inputs from the microenvironment, and promotes the dissociation of tumor cells from the primary site, with subsequent migration and dissemination to distant places. EMT programs are tissue-context dependent, and activated only partially and transiently, reflecting the balance of transcriptional drivers and suppressors of EMT [34,39,60,61,62,63,64,65]. These intermediate states represent crucial drivers of tumor progression [63]. The term ‘epithelial–mesenchymal plasticity’ (EMP), also referred to as partial EMT, or metastable EMT state describes the ability of cells to adopt E/M epithelial–mesenchymal features and to interconvert, reversibly, between intermediate states [60,65,66,67,68]. Thus, EMT status cannot be assessed exclusively based on a reduced number of markers, and both epithelial or mesenchymal traits and features, such as invasion, increased survival, or decreased proliferation, should be included as criteria for accurately defining EMT status. A common feature is an attenuation in the early phase of the epithelial phenotype [69,70,71,72,73]. Of note, tumor cells activating EMT acquire just a few mesenchymal traits, sufficient to confer the ability to invade adjacent tissues, and disseminate to distant sites [34,65,74,75,76]. The activation of alternative EMT programs and intermediate mesenchymal states generate great phenotypic heterogeneity within tumors and is proven to occur also in CTCs released by primary cancers and their metastases [77,78].

Cancers originating from the mesenchymal or neuronal lineages consistently present higher EMT scores, while solid tumors of epithelial origin have different EMT gradients, which might explain the unconvincing clinical significance of EMT in such cases [79]. Multiple tumor cell subsets associated with different EMT stages have been identified [61], reflecting heterogeneous levels of markers previously associated with tumor stemness, EMT, or metastasis initiation—such as CD61, CD51, and CD106 [80,81,82,83,84]. 

The epithelial cell adhesion molecule EpCAM is overexpressed in tumors of epithelial origin [85,86], and undifferentiated human embryonic stem cells [87]. In tumor cells, the dynamic expression of EpCAM correlates with the EMT and the reverse mesenchymal–epithelial transition (MET) processes since it is transiently lost during the early phase and subsequently re-acquired in metastatic niches [88], thus limiting the detection of epithelial–mesenchymal transitioned CTCs [89,90]. Conversely, the EMT process is associated with overexpression of vimentin [91], a constituent of adhesion networks, which is confirmed in single-cell profiling of CTCs [91,92,93] and directs CTCs to reseed the metastatic niche. Accordingly, CTCs were recently detected and enumerated from patients with epithelial colon cancer using cell-surface vimentin (CSV) [94]. CSV+ EMT CTCs might represent a unique subset of CTCs not responding to chemotherapeutic regimens, and could be associated with cancer stem-like cells, providing evidence that EMT CTCs detection is critical for patients with tumor progression. 

The hypothesis that full EMT is associated with increased metastatic potential and promotes initial steps of metastasis is still a matter of debate [34,61,63,95,96,97,98,99,100,101], despite a lot of evidence of EMT as a relevant switch in systemic cancer and treatment resistance [62,95,98]. On the other side, the metastatic potential has been proven to greatly correlate with the intermediate EMT state, and to less extent with the levels of expression of the CD106 metastatic marker [61]. In accordance, the majority of CTCs underwent EMT and expressed both epithelial and mesenchymal markers [77,95]. Of note, even the most mesenchymal states are not irreversibly committed, and cells can undergo MET within the metastatic microenvironment [102]. The distinct intermediate EMT states are associated with diverse invasive, metastatic, and differentiation characteristics: tumor cells with hybrid phenotypes more efficiently enter the circulation, and generate metastases, with implications in tumor heterogeneity, invasion, metastasis, and resistance to therapy [34,36,61,65,74,75].

EMT has been documented to confer resistance to cell death in tumors to chemo- and immunotherapy in experimental and clinical studies [32,96,97,103], and targeting EMT holds promise in overcoming therapy resistance. Earlier reports which addressed EMT in CTCs demonstrated a correlation between mesenchymal CTCs and therapy resistance [77,96,97]. The mesenchymal lineage is linked to enhanced escaping of anoikis and drug-induced death [103], which has been linked to drug susceptibility and to the entrance of tumor cells into a non-proliferative state in which they have stem-cell-like properties [104]. Thus, even if the functional implications of EMT heterogeneity are still unknown, several clinical trials have accepted the notion of EMT plasticity for the potential for targeted therapy to prevent cancer metastasis. 

Interestingly, CTC clusters, which are defined as a group of two or more distinct clonal carcinoma cells, have a greater metastatic potential and are more effective in colonizing secondary sites than single mesenchymal CTCs [52], thus highlighting that mesenchymal cancer cells require at least partially reverse to the epithelial state for metastatic growth [105]. Although representing a minority of the CTCs in the peripheral circulation, the presence of CTC clusters is associated with the worse clinical outcome in multiple cancer types [52,106,107]. CTC clusters have distinct gene expression profiles and disseminate differently compared to single CTCs. They retain epithelial features, with cell–cell junctions exerting a key function for their maintenance in the circulatory system [52,108,109,110]. Heterogeneous clusters might seed polyclonal metastases [108,111,112], thus increasing the likelihood that a tumor will colonize distant tissues and might eventually present increased resistance to therapies.

Numerous pieces of evidence in the literature reported the link between the acquisition of EMT and cancer stemness, characterized by an increase in tumor-initiating cells (TICs) frequency [65,102,113,114]. As recently reviewed, several lines of evidence coherently confirmed that the metastatic potential of a tumor is due to a low number of a minor subpopulation of cancer cells—termed cancer stem cells (CSCs)—able to self-renew and to efficiently regenerate the phenotypic heterogeneity of a parental tumor, and responsible for initiating overt metastases; of note, CTCs with stemness properties have been documented, which represent the most aggressive tumor cells in the circulation [55]. Activation of EMT is a major mechanism for the generation of cancer stem cells (CSCs), and TIC frequency increased at the earliest EMT state [102]. Different subsets of CSCs coexist, which present an EMT-derived stem cell phenotype, expressing a CD44highCD24low profile [114,115,116], or a high intracellular aldehyde dehydrogenase 1 activity, as a marker of stemness [117,118]. CSC-like properties and EMT features were both induced by the gene signature for the induction of pluripotency [119]. Cells with an intermediate mesenchymal–epithelioid state exhibit CSC-like properties [114,120,121,122,123,124]. Recently, a sub-set of metastasis-initiating cells (MICs) has been described among CTCs [56,125,126], Thus CTCs with a hybrid epithelial/mesenchymal phenotype present also stemness traits [61,77,127,128], which have been correlated to adverse disease outcomes and drug resistance [129,130]. Following recent literature [96,97], mesenchymal-type CTCs were more resistant to chemotherapy compared to epithelial-type CTCs. Systemic tumor cells with a hybrid phenotype, which is defined as primarily epithelial and moderately mesenchymal, represented the most aggressive cells, with enhanced ability to generate metastases, consistently with EpCAM function in cell adhesion, proliferation, and epithelial differentiation [131], and correlated with the occurrence of lung metastases in several types of cancer [101], such as in prostate, breast, bladder, and pancreatic carcinomas [96,125,126,132,133,134]. In a clinical cohort of patients with stages III and IV metastatic breast cancers, by using EpCAM as a marker for EMT, the loss of epithelial phenotype in CTCs has been demonstrated, while bone marrow-derived disseminated tumor cells retained an epithelial phenotype [118]. These results support the notion that, despite the low numbers [41,42], EpCAM+ systemic circulating tumor cells represent the primary source of MICs [125,126].

At present, for a clinical application of CTC [33,42], collected data suggest that EpCAM-dependent enrichment systems underestimate CTC numbers, even if without losing clinically relevant cells. Accordingly, EpCAM+ CTCs counts predict the clinical outcome of patients with metastatic and non-metastatic breast cancer [42,135]. 

Bone and STSs may reside in an intermediate metastable phenotype, shifting from a more proliferative (epithelial-like) to a more invasive (mesenchymal) state, which expresses both epithelial and mesenchymal markers. Comparative expression of epithelial markers and adhesion molecules indicated heterogeneous expression across types [136,137,138,139]. Accordingly, a strong variance in EpCAM expression has been documented between histotypes in pediatric sarcomas and the association with patient outcome [140,141]. The metastable EMT state is common in aggressive sarcomas and frequently contributes to drug resistance [142]. Osteosarcoma and desmoplastic small round cell tumors (DSRCT) have been documented to express both epithelial and mesenchymal markers, such as keratin, cadherins, desmin, and vimentin [143,144]. Due to deregulation of EMT-related genes, DSRCT presents high inter-tumor heterogeneity, frequently disseminates and resists drug treatment [145]. Similarly, in synovial sarcoma restricted areas of the primary tumor mass, as sites of spontaneous EMT, differentiate towards the epithelial lineage [146]. An epithelial/mesenchymal differentiation has been documented in Ewing sarcoma and epithelioid sarcomas too. Ewing sarcoma tumor cells with strong EWSR1-FLI1 transcription activity have an epithelial-like phenotype, whereas low-level EWSR1-FLI-positive cells mostly express cell-matrix proteins, have higher motility and invasive capacity but reduced proliferative activity [147]. Of note, MET-related programs occur in sarcomas expressing epithelial markers, such as E-cadherin and β-catenin in synovial sarcomas [146], or in Ewing/PNET tumors, which have high levels of tight junction proteins [139]. The prognostic significance of expression of an epithelial marker and adhesion molecule in sarcoma tumors has not been assessed systematically and only a few data have been reported. Desmoplakin and pGSK3β represent independent good prognostic factors for PFS, while ZO-1 and Snail are independent good prognostic factors for OS [138]. An active role of MicroRNA (miRNAs) (i.e., miR-130a, miR-126, miR-145) in the modulation of cadherins and epithelial cell markers has been reported, which determine a shift from a proliferative state to a more invasive phenotype of osteosarcoma cells [148]. Regulation of EMT/MET-related genes in rhabdomyosarcoma (RMS) tumors has also been described, mediated by activation of the Rac1/Cdc42-PAK signaling pathway, with loss of E-cadherin and expression of N-cadherin [149]. RMS cells express the lowest levels of EpCAM and E-cadherin among all sarcoma histotypes due to PAX3-FOXO1 transcription factor, which antagonizes wild-type Pax3-induced cell aggregation and epithelioid changes.

A dynamic expression of EpCAM during tumor evolution has been observed in diverse sarcoma subtypes, with a correlation between high EpCAM expression and significantly poorer patient’s OS and adverse outcome [141]. In patients with RMS, no significant difference in EpCAM expression has been observed between the onset at diagnosis and relapse, during the follow-up. However, patients with higher levels of EpCAM had a significantly poorer outcome, while EpCAM levels were not significant for PFS. Similarly, patients with DSRCT overexpressing EpCAM had a poor prognosis and OS. Thus EpCAM-positive tumor cells might exert a role in metastasis in sarcoma. It has been proven that EpCAM expression can be acquired during tumor progression [150] and is highly expressed in cancer stem cells [151]. The numbers of CTC-positive in patients with sarcoma and total CTCs in pediatric sarcomas are similar to data previously reported at diagnosis in carcinomas [152], with progressive disease and poor outcome in patients with the highest EpCAM levels [141].

## 3. Technologies for Detection of CTCs in Sarcoma

**Figure 1:**
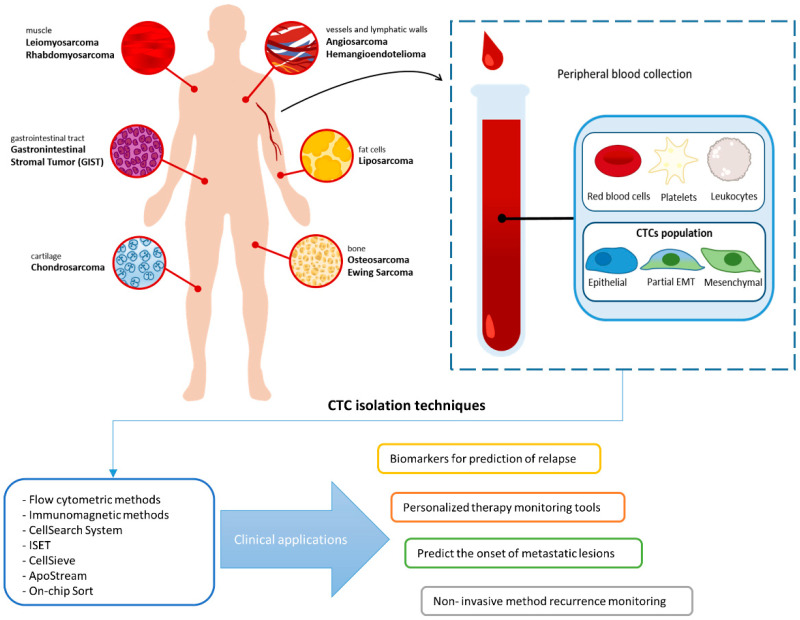
Technologies used to detect and isolate circulating tumor cells (CTCs) in sarcoma and their potential applications.

Recent papers report novel technical advancements in assaying for the presence, isolation, and enumeration of CTCs in patients with sarcoma, and several technologies have been described, based on imaging, microfilter, and microchip devices [89,90,153,154]; even if some studies still represent proof-of-concept. Standardization of methodologies is important to test the clinical utility of CTCs in prospective clinical trials for the identification of therapeutic targets and resistance mechanisms of systemic therapies, and patient stratification [155]. Few studies have been performed on sarcoma-derived CTCs (Figure 1), while in the majority of cases tumor cells have been detected by using PCR-based methods in peripheral blood and/or bone marrow, in Ewing sarcoma [156,157,158,159,160], RMS [161], synovial sarcoma [146], osteosarcoma [162], and alveolar sarcoma [163,164], analyzing tumor-specific markers (i.e., translocations [165], or by gene expression analysis [163,166]), with several limitations. Reports on the identification of mutations in ctDNA in predefined cancer genomic hotspots in sarcoma have been published [167,168], by improved sequencing techniques [169], providing a highly sensitive tool to detect mutations, for assessment of therapy response in metastatic patients with poor prognosis [170]. However, currently, the number of actionable mutations and defined drug-sensitivity networks is relatively low. In addition, mutations in the ctDNA may not be the endpoint of tumor transformation and may derive from apoptotic cells more sensitive to antitumor therapies. Additionally, ctDNA remains difficult to be detected in pre-metastatic patients. MicroRNAs have also been studied in sarcoma [171,172], although their utility in clinical practice has not been yet demonstrated.

Although few researchers reported the detection of transitioned CTCs using the existing technologies from patients with mesenchymal malignancies [77,92,173,174,175,176], the uncertainty calls for the discovery of novel markers for EMT CTCs. Mesenchymal markers, such as N-Cadherin, frequently present a relevant expression in peripheral blood mononuclear cells (PBMC) and, are not suitable to detect CTC and predict metastasis occurrence [177]. Recently, CTCs were detected and enumerated from patients with epithelial colon cancer using CSV [94]. A cut-off of <5 or ≥ 5 EMT CTCs has been defined as the threshold concerning therapeutic response, with a predictive value for the therapeutic outcome of 87%. 

It has been proven that EpCAM expression can be acquired during the progression of tumors [150] with a high expression in cancer stem cells [151]. By using the CellSearch™ System for CTC capturing, CTCs have been detected in the peripheral blood of patients with pediatric bone sarcoma and STS, which are not of hematopoietic origin [141]. CTCs were assayed in 11 patients at diagnosis before starting the therapy, by using epithelial and mesenchymal markers to capture CTCs, such as cytokeratins (CK8/18/19) and Desmin. Among sarcoma patients, 64% present at least one CTC per 7.5 mL, while 5 out of 11 (45%) patients had at least two CTCs, resembling data previously reported for carcinomas [152]. Moreover, 4 (57%) CTCs-positive patients had one Desmin-positive CTC, thus expressing markers of either an epithelial or mesenchymal phenotype [150].

In 2005, the earliest study has been performed to assay the validity and usefulness in clinics of an immunomagnetic method for detection of tumor cells in bone marrow (BM) and peripheral blood (PB) of patients with osteosarcoma, through testing of two osteosarcoma-associated antigens, namely a cell surface antigen with homology to the bone isoenzyme of alkaline phosphatase and the high molecular weight melanoma-associated antigen 9.2.27 [178]. Micrometastatic osteosarcoma cells have been isolated and enumerated, allowing rapid screening of 2 × 107 mononuclear cells with high sensitivity in BM (63% of the patients were positive), but with a low detection rate of tumor cells in the blood (8%). A high number of live cells rosetted with magnetic beads has been isolated, and the expression of proteins, with clinical and biological relevance, expressed on tumor cells, such as antigens used as therapeutic targets, or prognostic markers, e.g., HER-2/neu and MDR, can be analyzed [156,179]. A high fraction of bone marrow samples were positive at diagnosis, both in patients without and with metastatic disease, as detected with conventional diagnostic procedures. Of note, a higher fraction of patients with micrometastatic cells in BM relapsed concerning patients with negative samples [178].

In 2008, the presence of metastatic cells from patients with RMS was assessed in BM and PB by a flow cytometric method [180]. To identify tumor cells, a panel of antigens, i.e., CD45, CD56, CD90, and CD57, was used, and the RMS-specific transcript Myogenin (Myf4) [181] was molecularly detected. All seven BM samples from RMS stage IV presented the CD45– CD56+ phenotype and expressed the Myf4 transcript. Furthermore, four cases were positive also for CD90 and two for CD57. Neither the CD45– CD56+ phenotype nor Myf4 has been recorded in patients with localized disease. No circulating RMS cells were detected at diagnosis in the seven high-grade RMS patient samples, except for a PB sample collected on the progression of the disease, but only 100 μL of plasma were tested in flow cytometry. Since detection of the CD45– CD56+ phenotype by flow cytometry in BM aspirates has been recommended for staging or diagnosis in neuroblastoma stage 4 [180,182], similar detection in BM could be used only for the staging of diagnosed patients with RBM. Thus, these findings suggest that, since flow cytometry identified circulating cells in the PB from a subset of patients with neuroblastoma, but not of patients with stage 4 RMS, PB might be tested for non-invasive diagnosis, and flow cytometry may be a method to effectively detect RMS metastasizing cells [180].

In 2009, PBMCs of patients with bone sarcomas were analyzed by flow cytometry for mesenchymal stem cells (MSCs) phenotype, demonstrating an >9-fold increase in the number of cells in patients compared with control subjects [183]. Enumerated MSC-like cells were positive for CD44, CD90, and CD105 and negative for CD14, CD34, and CD45, as markers of hematopoietic cells [184]. Also, a higher level of plasma hepatocyte growth factor (HGF) [185] and vascular endothelial growth factor (VEGF) [186], mediating systemic mobilization of MSC-like cells into the peripheral circulation [187], have been measured [183]. The increased number of MSC-like circulating cells and elevated plasma concentration of HGF and VEGF may be used for diagnosis or prognosis in patients with bone sarcoma. 

In a relevant study in 2010, Dubois et al. [157] were the first to enumerate CTC in a patient with Ewing sarcoma through flow cytometric analysis of PBMCs, 75% of patients with Ewing sarcoma present with clinically localized tumors [131] and micrometastatic disease since the great majority of patients experienced recurrence [188]. To test the proof-of-concept of the method, cells with the CD99+CD45− profile were detected in both blood (0.0021%) and bone marrow (0.048%) of a subject with newly diagnosed localized Ewing sarcoma [157]. The presence of CD99+CD45− mononuclear cells in the bone marrow from patients without Ewing sarcoma at a higher rate compared to PBMCs, representing early monocytic progenitor cells [189,190], demonstrate at present no sufficient specificity of BM testing for Ewing sarcoma diagnosis. Clinical evaluation and validation of the method are ongoing since follow-up indicated that patients with clinically non-metastatic tumors have detectable EWS fusion transcripts in PB and/or BM and may have an inferior outcome [156]. Flow cytometry for detection of CD45 and CD99 circumvents the need of RT-PCR technique of prior knowledge of EWS fusion oncogene present in the tumor for CTCs analysis. A clinical study to determine the ability of the methodology to detect CTCs in patients with the newly diagnosed or relapsed disease is ongoing.

In 2014, to detect and enumerate mesenchymal CTCs from sarcoma tumors, irrespective of the origin, a monoclonal antibody directed against the cell-surface vimentin protein (CSV) has been generated, while not recognizing the intracellular vimentin expressed in normal mesenchymal cells, including the majority of white blood cells [92]. Upon CD45 depletion and CSV–positive selection, cells were recovered and subjected to immunofluorescence staining and detected by flow cytometry. An increase in CTC count was observed in metastatic patients at the time of the first clinical presentation; also, CTC counts were lower in patients previously subjected to chemotherapy. Once isolated, CTCs were further characterized by single-cell mutation analysis or fluorescence in situ hybridization (FISH). In angiosarcoma, mutations in TP53, and not in FLT4, were detectable only in CTCs, concerning the primary tumor, thus confirming cell heterogeneity [191]. Osteosarcoma-derived CTCs were assayed for MDM-2 and KRAS amplification, highlighting a difference between patients diagnosed with metastasis in the lung or localized osteosarcoma [192,193], thus predicting the onset of metastatic lesions at distant sites and potentially the therapeutic efficacy of drugs [92]. This is the first proof-of-principle study to enumerate and validate CTC from different sarcomas using a single marker, providing a prognostic tool to monitor cancer metastasis and relapse. 

The cytometric technique for sarcoma CTCs capture and detection has been validated also using cryopreserved PBMCs preparations from patients with multiple sarcoma and CSV as a specific target [194]. EMT-like CTCs have been captured and enumerated with high sensitivity and specificity [92,142,195]. The technology will enable the bio-banking of samples to be processed in large numbers and will improve the CTC-based diagnosis and treatment in clinical settings [194].

The minimal residual disease (MRD) assessment is of the utmost importance to evaluate the risk of metastasis and treatment efficacy. In advanced localized RMS, recurrence is common, and the prognosis is poor [161]. RMS is thought to originate from myogenic precursors expressing PAX3 [196], thus—due to its expression in most RMS tumors [197]—PAX3 has been tested in flow cytometry as a marker for circulating RMS cells, even if not for differential diagnosis since its expression has been described also in Ewing sarcoma [198]. PAX3 is expressed in all RMS tumors analyzed, thereby pointing to PAX3 as an additional MRD marker to detect circulating or disseminated disease [199]. Of note, PAX3 levels were heterogeneous in RMS, higher in alveolar (aRMS) than in embryonal (eRMS) subtype. The usefulness of flow cytometry has thus been confirmed as a more specific and reliable tool for MRD assessment in RMS than qPCR, especially due to its greater sensibility.

Recently, the potential role of nuclear-programmed death-ligand 1 (PD-L1) expression in vimentin-positive CTCs, has been assessed as a clinically relevant prognostic marker in tumors [195], thus detecting epithelial–mesenchymal transitioned CTCs. Aberrant expression of PD-L1 has been documented in several cancer types [200,201,202] and clinical trials are ongoing to assess the prognostic relevance of PD-L1 [203]. CTCs were enumerated also in osteosarcoma and PD-L1 expression has been analyzed using confocal microscopy. PD-L1 was detectable in CTCs and localized in the membrane, cytoplasm, and nucleus, in the majority of cells [195].

A method for the enumeration of CTCs, based on abnormal chromosome numbers (aneuploidy) in CTCs, was recently developed and validated in a prospective cohort of patients with primary and recurrent/metastatic osteosarcoma subjected to surgery [204]. Accordingly, CTCs were characterized by FISH and immunocytochemistry for cytokeratin and CD45, in order to exclude epithelial and lymphocytic cells, respectively. Patients with osteosarcoma with ≥2 CTCs per 7.5 mL of PB had significantly shorter PFS than patients with <2 CTCs. The FISH technique represents a reliable tool for the quantification of CTCs and can be clinically standardized. 

Flow cytometry-based technologies for detection of sarcoma CTCs [9,157,205,206] present some limitations, for instance, the lack of a histology-specific marker and the heterogeneous expression of target markers, while CD99 is expressed at a low extent also on cells of the B lineage [189], which may reduce the detection sensitivity. Also, the use of flow cytometry requires extensive processing of cells, and CTC clusters are not detected due to gating on single cells [52,207]. Thus, other technologies have been applied for CTC capture.

Some authors have addressed the sensitivity and specificity of the ‘isolation by size of tumor cells’ (ISET) diagnostic technique, for CTC enrichment and detection [208,209]. This method relies on the tumor cellular size which is larger than leukocytes, thus isolating also CTCs in epithelial–mesenchymal transition [210,211]. CTCs have been detected in patients with metastatic/recurrent or locally advanced sarcomas. Cells were identified by cytomorphology and further characterized by immunocytochemistry with anti-vimentin or anti-Pan CK, and anti-CD45, to distinguish them from endothelial cells, leukocytes, and epithelial cells [212]. Of note, circulating tumor microemboli (CTM), which are a marker of poor prognosis [213], have been also identified. The ability and sensitivity of the ISET method have been previously assessed in sarcoma cells [208,214], and Hofman et al. [215] reported the detection of CTCs from patients with sarcoma. In Chinen et al., CTCs have been reliably identified and counted in all patients, and CTC enumeration could be used in patient follow-up, to monitor therapy for personalized medicine [212]. Vimentin has been detected in CTCs from STSs, which is associated with EMT, stemness, and more malignant characteristics of these tumors [36,214,216]. Different types of sarcomas have been assayed, the majority of which are not characterized by genetic mutations or fusion transcripts, thus not detectable through a PCR-based test. Also, ISET allows the study of CTC morphology and cell immunomolecular characterization. Future studies are required to correlate its expression with the clinical outcome of patients.

The CellSieve™ size-exclusion low-pressure microfiltration system [217] has been proven an effective method to efficiently collect CTCs in several carcinomas [111], with a potential application in monitoring disease response and prediction of metastatic relapse. Its utility has been confirmed also in patients with high-grade sarcomas of different histology [217]. This approach has several benefits since cells are minimally processed and a sarcoma-specific cell surface antigen is not required. Sarcoma CTCs were defined as positive for vimentin, negative for CD45, and with a nuclear morphology that is distinct from normal white blood cells. Both epithelial and mesenchymal types of CTCs have been captured, with high sensitivity, and accurately quantified. Additionally, CTC clusters have been identified and enumerated, more frequently in patients with a newly diagnosed disease or metastases. CTC-derived RNA was analyzed, demonstrating an EWS-FLI1 translocation and identifying a previously unrecognized p53 mutation in Ewing sarcoma, and single-cell RNA sequencing in aRMS. Twenty-eight differentially expressed genes positively identify the cells in the cluster, being part of a previously published aRMS expression profile or expressed in either the myogenic lineage or in RMS cells. CTCs have been collected after minimal manipulation, for molecular analyses for either diagnostic or research purposes.

The ApoStream™ CTC isolation device has been designed by the National Cancer Institute as a clinically-suitable technology for the capture of rare tumor cells and the analysis of molecular biomarkers of pharmacodynamics response to drug therapy. It uses dielectrophoresis principles to separate cells—i.e., non-hematopoietic cells from PBMC fraction—with distinct morphological and biophysical properties—i.e., membrane capacitance, morphology, size, electrical conductivity [218,219,220]. Upon isolation, CTCs from patients with Alveolar Soft Part Sarcoma have been efficiently identified using a multiplex immune-phenotyping assay to CTC biomarkers vimentin (VIM), cytokeratin (CK), and β-catenin (β-cat), while CD45+ cells have been excluded, to increase the confidence of cell identification and potentially include CTCs from different sarcoma [221]. The great majority of cells presented the CD45−/(CK/β-cat)−/VIM+ phenotype. The identity of enriched tumor cells has been proven through the detection of the hallmark ASPL-TFE3-T1 translocation [222]. Data demonstrated the heterogeneity of CTC phenotypic profiles: of note, at baseline, the epithelial phenotype has been evidenced in CTC by the presence of a CD45−/(CK/β-cat)+/VIM-subset. This study is an initial proof of research and clinical reliability of the ApoStream™ instrument, which will facilitate isolation of viable CTC, monitoring over time of drug effects, and disease management for patients with sarcoma [221].

On-chip sort is a cell sorter on a microfluidics chip that includes a collection reservoir to reliably collect the target cells without loss [223]. A protocol has been established for the collection and molecular profiling of CTCs from sarcoma [224]. A multi-gene panel test was used to confirm somatic mutations present in the tumor of origin. CTCs have been enriched by blood cell depletion using CD45 and CD235a MicroBeads. The remaining cells were fixed and stained to detect vimentin, which is specific for sarcoma, or CD45 and CD14, to detect white blood cells. Then CTCs, represented by the CD45−/vimentin+ fraction, have been enumerated and sorted using the on-chip sort system. By improving the sorting method, CTCs can be identified in patients before metastasis. In a pilot study, CTCs were recovered from a patient with locally advanced myxofibrosarcoma. The nonsynonymous mutation for KMT2B p.Ile2602Val was identified in the tumor biopsy and confirmed in CTC. The clinical utility of the current protocol to laboratory testing for monitoring early metastasis and recurrence and molecular profiling of tumors and for decision making must be tested in larger cohorts.

## 4. Clinical Significance of CTC in Sarcoma Tumor

Clinical trials have demonstrated that the presence of detectable CTC is associated with poor prognosis in carcinoma [225,226,227,228]. Preliminary studies showed a trend [166] or clear evidence [156,160] of the prognostic value of CTC detection even in sarcoma. However, research regarding the utility of detection of CTCs in sarcoma remains insufficient and only a few clinical trials have been set on this item (as listed in Table 1) [229]. Briefly, results on CTCs assessment have been reported in just two out eight studies, which have been terminated. In a first study (NCT00474760), in order to monitor the response to Anti-IGF-IR CP-751,871, both total and Insulin-like Growth Factor 1 Receptor (IGF-1R) Positive CTCs were quantified using an automated microscope system; however cells were detected in an insufficient number of patients to confirm any clinical validity. In the second study (NCT02783599), a reduction in the number of CTCs, measured through a fluorescence scanning method, has been reported in patients responsive to at least one cycle of olaratumab as monotherapy [230].

In preliminary results of Satelli et al. [92], MDM-2 and KRAS amplification has been assayed in osteosarcoma-derived CTCs; these genomic alterations have been reported in lung metastasis, while absent in localized tumors. Since MDM-2 and KRAS amplifications have been previously documented in metastatic osteosarcoma [193], detection of this amplification in CTC could predict the onset of distant metastatic lesions.

Single-cell analysis of CTCs has recently provided evidence of the existence of aneuploid CTCs [231]. Aneuploidy is defined as an unbalanced chromosome content and is a hallmark of cancer [232], associated with altered gene expression profiles, increased metastatic potential, resistance to treatments, and overall poor prognosis [233,234]. In a prospective cohort of surgical patients with primary and recurrent/metastatic osteosarcoma (n = 23), the number of CTCs, identified by aneuploidy, as assayed in FISH, were compared in patients with primary, recurrent or metastatic osteosarcoma: patients with ≥2 CTCs per 7.5 mL of PB had significantly shorter PFS than patients whose PB contained <2 CTCs [204]. CTC enumeration in patients with osteosarcoma has prognostic value in both primary and metastatic tumors, since the presence of CTCs is associated with poor clinical outcome [204], it is minimally invasive to recover samples and it is required a simple protocol to enumerate cells. Moreover, it may be utilized as a personalized therapy monitoring tool to select effective treatment strategies in early and advanced osteosarcoma. However, the validation of the sensitivity and specificity of the method and its prognostic power should be further addressed in a multi-center prospective clinical trial with larger patient cohorts.

Aneuploid CTCs have also been identified in STS patients, in a pilot study in 2020; FISH analysis has been performed and CTCs were identified in all four metastatic STS patients tested, with a median value of 4 per 7 mL of blood [235]. In silico evidence using data from STS cohort of The Cancer Genome Atlas Project and the validated Aneuploidy Score confirmed the prognostic role of aneuploidy in mesenchymal cancers, with a significantly worse PFS and OS in the group with a high DNA aneuploidy, independently of the histology of the tumor. Coherently, a transcriptional signature of 67 genes related to mitosis and chromosome integrity (CINSARC) has prognostic potential for clinical outcome in STS patients [236]. The method used is agnostic for the expression of surface markers, thus CTCs in different stages of their epithelial–mesenchymal or mesenchymal– epithelial transitions could be identified. The longitudinal evaluation of the number and ploidy of CTCs might represent a novel tool to evaluate STS patients’ prognosis and response to treatment and is currently under prospective evaluation.

In metastatic sarcoma patients, the prognostic impact of CTCs and CTM—and the expression of EGFR in these cells, which are associated with reduced survival in solid tumors [237]—has been analyzed before chemotherapy [238]. CTCs were detected by ISET technology in 94.4% of patients, with a median number of 2.0 CTC/mL (0–11 CTCs/mL). The presence of CTCs indicates inferior median PFS in patients, but without statistical significance. CTMs have been detected in 27.7% of patients, with a lower median PFS and OS compared to those with no CTMs, independently of the therapeutic regimen. EGFR protein was expressed in CTC in the majority of patients (94%). A possible correlation of EGFR-expressing CTCs with poor prognosis and clinical outcome has been reported, even if not statistically significant. EGFR has been proven to be highly expressed in STS tissues, and associated with high histological grade [239,240]. In a recent study, certain sarcoma subtypes have been hypothesized to reside in a ‘metastable’ state expressing both epithelial and mesenchymal features, with activation of reversible EMT/MET related programs, individual tumor cells acquiring the characteristics of more differentiated cells in response to specific stimuli [142]. This molecular heterogeneity could lead to clinically highly aggressive cells, with important clinical implications. EGFR activity enhances tumor growth, invasion, and metastasis, thus conferring a more aggressive phenotype, and further supporting the epithelial–mesenchymal plasticity model in patients with sarcoma: indeed invasion and dissemination in non-epithelial tumors could be due to EGFR expression on CTCs. These results point to EGFR as a new target in STS treatment [238]. CTC, CTM, and EGFR expression can be reliable tools to measure the therapeutic effectiveness and to select patients for clinical intervention, requiring studies with a larger cohort of patients, defining treatment options and follow-up time points to confirm data.

Recently, CTCs have been isolated from 18 patients diagnosed with Ewing sarcoma, based on the immunomagnetic separation of CD99-positive tumor cells [241]; EWSR1/FLI1 or EWSR1/ETS-related gene transcripts have been confirmed through quantitative and digital RT-PCR on cDNA obtained from CTCs, with a limit of detection of 1 cell/mL of PB. PB samples were collected randomly during therapy, and from patients further hospitalized, thus 23 samples were processed according to the CTC immunoseparation protocol and analyzed in RT-qPCR and RT-dPCR. CTCs have been detected in patients with Ewing sarcoma, independently of the specific molecular rearrangement. Patients scored positive for CTC and RT-PCR testing at diagnosis, while resulting negative after chemotherapy. These techniques potentially improve risk stratification and early response assessment, for prognostic and predictive purposes in Ewing sarcoma. In this study, the CTCs assay was proven to reliably detect the amplification of EWSR1/FLI1 and EWSR1/ERG transcript fusion genes. Isolation of CD99-positive CTCs will help identify the metastatic precursor cells and direct novel diagnostic therapy and prognostic options upon therapeutic monitoring of patients with Ewing sarcoma, for clinical decision-making. A clinical study to detect tumor cells in the blood and bone marrow of patients with Ewing sarcoma, with newly diagnosed or relapsed disease, by using the methodology described is ongoing.

Currently, chemotherapy in osteosarcoma consists of four alkylating agents: high-dose methotrexate with leucovorin rescue, doxorubicin, cisplatin, and ifosfamide [92,242]. Frequently therapeutic response is low, with the development of lung metastases, highlighting the need to identify early biomarkers to detect recurrence and metastasis. CTCs have been detected in a pre-clinical model of human osteosarcoma by using the DEPArray technology, and the kinetics of release of CTCs and their modulation after chemotherapy were monitored over time [243]. CTCs were detectable at an early stage, thus suggesting that CTCs represent a non-invasive method to monitor the recurrence in osteosarcoma. Of note, at the early stage, one cycle of ifosfamide decreased the number of lung metastases, while a significant increase in CTCs number has been measured. At a later stage, one or two cycles of ifosfamide, independently of the dose regimen, reduced the growth of primary tumors and did not modulate CTC count, which was higher than the early stage of the disease, thus pointing to a potential ‘equilibrium’ in osteosarcoma in the metastatic process. However, detectable CTCs after ifosfamide therapy generated lung metastases less efficiently. Accordingly, CTC heterogeneity determines their extravasation capability into the metastatic site, to give metastatic nodules [50,244]. The number of CTCs may reflect the response to therapy in patients with osteosarcoma. The next step of this proof-of-concept study will be to determine the value of CTC in osteosarcoma, both from a biological and clinical setting, thus it will be required to enumerate and characterize CTCs in a large series of metastatic and non-metastatic patients and to assess the kinetics of CTC release during chemotherapy.

Using a modified immunomagnetic method [92,245] and CSV as a circulating cell capturing tool through immunofluorescent imaging, a novel class of CTCs, positive for CSV and macrophage-like (ML), have been identified in GIST. These cells have been defined as macrophage-like, expressing macrophage markers CD14 and CD68 and tumor markers C-kit, DOG-1, and CSV, and negative for CD45 [246]. Tumor-associated macrophages (TAMs) originate from the microenvironment within several primary tumors and have a key role in tumor invasiveness and immune suppression prediction [247]. CTCs extravasate capillaries and, anchored to CTC-educated TAMs, set up secondary metastatic lesions, with increased tumor invasiveness [246,248]. A cluster of CTCs and circulating TAMs have been detected in different progressive and metastatic tumor types [92,142,245]. Significantly greater numbers of CSV-positive ML-CTCs were detected in patients with metastatic than localized GIST (*p* < 0.0001) [246], thus acting as a novel biomarker for prediction of relapse/metastasis in patients with GIST. Upon validation in large sample cohorts, ML-CTCs could be used in clinics for diagnosis of metastasis and relapse of GIST.

A strong correlation has been demonstrated between EpCAM expression level and increased risk of relapse and lower OS in several prospective clinical studies [41,249,250]. By using the CellSearch™ platform, CTCs have been detected in the peripheral blood of pediatric patients with sarcoma, they are not hematopoietic cells and express markers of epithelial or mesenchymal phenotype [141]. In 8 out of 11 patients analyzed, EpCAM expression was assessed in the primary tumor too. Of note, two patients with metastasis at diagnosis (high-grade RMS) showed detectable CTCs and were positive for EpCAM expression in tumor tissue. Among five patients who progressed during tumor evolution, four were positive for tissue EpCAM expression and three had detectable CTCs at diagnosis. Two out of these three patients had a fatal outcome.

By using CellSieve™ technology, CTCs have been detected at diagnosis in most patients with newly diagnosed high-grade sarcoma, including Ewing sarcoma, osteosarcoma, dedifferentiated liposarcoma, RMS, synovial sarcoma, DSRCT, chondrosarcoma [217]. Although the number of cells detected was relatively low in 11 of the 18 patients (26 or fewer CTC and/or clusters), five patients presented higher numbers of CTC and/or clusters. Of clinical relevance, CTC quantification precedes clinical symptoms and detection of relapse through a radiographic test, before the development of overt metastasis, thus allowing early detection of metastatic recurrence. Indeed, three out nine patients with no radiographic evidence of tumor at sample collection presented CTCs, and all relapsed within 1–2 months. Further, dynamic changes in CTC numbers have been analyzed due to therapeutic response: samples were collected at several time points from 10 patients, all of them had detectable CTC and CTC clusters at the initial time. Upon therapy, CTC became undetectable in six patients, and five of these were in remission. Three of the four patients with detectable CTC over time relapsed. This proof of principle study demonstrated that sarcoma CTC can be reliably detected, accurately quantified, and collected; a reduction in CTC number correlated with response to therapy. Also, the presence of CTC identified patients relapsing, even if with documented radiographic remission, confirming the prognostic potential as biomarkers of MRD in sarcomas. Clinical trials of maintenance therapy should be designed accordingly.

## 5. Conclusions

In the present review, the clinical relevance of studying intact CTCs in sarcoma has been discussed, and weaknesses encountered in recovering viable mesenchymal tumor cells, cells with epithelial–mesenchymal intermediate phenotypes and cancer stem cells into the circulation of patients have been described, further highlighting the need for the scientific community to solve this item to offer a more reliable approach based on liquid biopsy to monitor sarcoma in the clinical setting. Preliminary results, as presented in the last paragraph—even if most preliminary in small patients cohorts—hold a great promise in this direction for individualized therapy of patients with sarcoma, and, hopefully, CTC enumeration and characterization would be added in clinical practice as an additional standard for monitoring over time tumor evolution. Technological advancement will further improve the sensitive and reliable identification and isolation of viable sarcoma cells, holding a great clinical value for initial staging of the tumor, for early detection of relapse, to improve prognosis and predict response to chemotherapy, and for identification of non-responders to therapeutic interventions. For instance, CTCs assessment could be complemented with the most innovative imaging techniques in STS in order to both improve non-invasive evaluation of response to neoadjuvant therapies and define tumor heterogeneity. Ultimately these studies may uncover the mechanisms of aggressiveness and metastatization in sarcoma for research purposes, with the potential to identify novel therapeutic strategies.

Despite the promising results discussed above, CTC assessment obviously still present some limitations for a reliable application in clinical practice [227,251]. For instance, capturing viable/whole CTCs from peripheral blood is demanding, and strictly depends on the technology used for CTC isolation or enrichment. Indeed, the selection of markers for CTC detection is a key question, since it affects the pool of cells which might be recovered with the available methodologies described in the literature. Furthermore, CTC assays need more standardization, as recommended by international consortia, including the European Liquid Biopsy Society (ELBS, www.elbs.eu, 1 February 2021). In addition, in early stage patients frequently low amounts of CTCs circulate in the peripheral blood, thus limiting the rate of recovery of tumor cells. Furthermore, the knowledge on the biology of the heterogeneous CTCs in sarcoma and their significance is still preliminary, thus requiring further studies in order to be reliably transferred to the bedside to improve their clinical use. 

Additional biomarkers in liquid biopsy are, at present, matter of investigation for both research and clinical feasibility in sarcoma. Indeed, great interest has been directed in these last years in addressing multiple biomarkers in the circulation of cancer patients at the same time, for clinical practice, for instance circulating exosome, CTCs, and nucleic acids, deserving a revision of the most recent literature on this topic. 

## Figures and Tables

**Table 1:** Clinical trials including detection of CTCs in sarcoma registered at https://www.clinicaltrials.gov, 1 February 2021.

Clinical Trial ID.	Title	Status	Study Results	Tumor Types	Clinical Intervention	Phase	No. Subjects	Study Type
NCT02983539	Detection of Circulating Tumor Cells in Patients with Sarcomas	Unknown status	No Results Available	Leiomyosarcoma, Pleomorphic Liposarcoma, Synovial Sarcoma, Liposarcoma			20	Observational
NCT03357315	Mix Vaccine for Metastatic Sarcoma Patients	Completed	No Results Available	Metastatic Sarcoma	Biological: Mix vaccine	Phase 1, Phase 2	30	Interventional
NCT02849366	Combination of Cryosurgery and NK Immunotherapy for Recurrent Sarcoma	Completed	No Results Available	Recurrent Adult Soft Tissue Sarcoma	Device: Cryosurgery Biological: NK immunotherapy	Phase 1, Phase 2	30	Interventional
NCT02783599	A Study of Olaratumab (LY3012207) in Participants with Soft Tissue Sarcoma	Completed	Has Results	Soft Tissue Sarcoma	Drug: Olaratumab, Doxorubicin External Beam Radiotherapy	Phase 1	51	Interventional
NCT04239443	Clinical Study of PD-1 Monoclonal Antibody SHR-1210 and Apatinib in Advanced NSCLC, Soft Tissue Sarcoma, and Uterine Cancer	Recruiting	No Results Available	Advanced Non-Small Cell Lung Cancer, Uterine Cancer, Soft Tissue Sarcoma	Drug: PD-1 inhibitor, Apatinib	Phase 2	120	Interventional
NCT01831609	Biomarker Analysis of Solid Cancers Such as Gastrointestinal Cancer	Recruiting	No Results Available	Sarcoma			1000	Observational
NCT04628806	Heat Shock Protein (HSP) 70 to Quantify and Characterize Circulating Tumor Cells	Not yet recruiting	No Results Available	Melanoma Stage IV, Sarcoma, Squamous Cell Carcinoma, Pancreatic Cancer Stage IV, Prostate Cancer, Breast Cancer Stage IV	Diagnostic Test: CTC isolation by HSP70		120	Observational
NCT03011528	First-line Treatment of Ewing Tumors with Primary Extrapulmonary Dissemination in Patients From 2 to 50 Years	Recruiting	No Results Available	Ewing Sarcoma Family of Tumors	Drug: VDC-IE x2, VDC-IE, TEMIRI, BuMel Local treatment by surgery or radiotherapy	Phase 2	45	Interventional
NCT03818412	Circulating Tumor DNA in Soft Tissue Sarcoma	Recruiting	No Results Available	Soft Tissue Sarcoma	Procedure: tumor tissue collection and blood draws	Not Applicable	40	Interventional
NCT02859415	Continuous 24h Intravenous Infusion of Mithramycin, an Inhibitor of Cancer Stem Cell Signaling, in People with Primary Thoracic Malignancies or Carcinomas, Sarcomas or Germ Cell Neoplasms with Pleuropulmonary Metastases	Recruiting	No Results Available	Esophageal Neoplasms, Lung Neoplasms, Mesothelioma, Thymus Neoplasms, Neoplasms, Germ Cell, and Embryonal	Drug: Mithramycin	Phase 1, Phase 2	60	Interventional
NCT00474760	Study of Anti-IGF-IR CP-751,871 In Patients With Solid Tumors	Completed	Has Results	Sarcoma, Ewing’s	Drug: CP-751,871	Phase 1	65	Interventional
NCT03085225	Trabectedin Combined with Durvalumab in Patients with Advanced Pretreated Soft-tissue Sarcomas and Ovarian Carcinomas.	Active, not recruiting	No Results Available	Ovarian Carcinoma, Soft Tissue Sarcoma	Drug: Combination of trabectedin with durvalumab	Phase 1	50	Interventional
NCT02636725	Axitinib and Pembrolizumab in Subjects with Advanced Alveolar Soft Part Sarcoma and Other Soft Tissue Sarcomas	Active, not recruiting	No Results Available	Alveolar Soft Part Sarcoma, Soft Tissue Sarcomas	Drug: Axitinib, Pembrolizumab Blood Draw, Tumor Specimen Collection	Phase 2	33	Interventional
NCT03946943	Study of Anlotinib Hydrochloride and Toripalimab in Subjects with Unresectable or Metastatic Undifferentiated Pleomorphic Sarcoma	Not yet recruiting	No Results Available	Soft Tissue Sarcomas, Undifferentiated Pleomorphic Sarcoma	Drug: Anlotinib, Toripalimab Blood Draw, Tumor Specimen Collection	Phase 2	25	Interventional
NCT01528774	Culture and Characterization of Circulating Tumor Cells (CTC) in Melanoma and Other Cancers	Completed	No Results Available	Melanoma	Blood Draw		150	Observational
NCT03600649	Clinical Trial of SP-2577 (Seclidemstat) in Patients with Relapsed or Refractory Ewing Sarcoma	Recruiting	No Results Available	Ewing Sarcoma	Drug: SP-2577	Phase 1	50	Interventional
NCT04052334	Lymphodepletion Plus Adoptive Cell Therapy with High Dose IL-2 in Adolescent and Young Adult Patients With Soft Tissue Sarcoma	Recruiting	No Results Available	Sarcoma	Drug: TIL, Interleukin-2, Fludarabine, Cyclophosphamide	Phase 1	15	Interventional
NCT01222767	Study of Zalypsis (PM00104) in Patients with Unresectable Locally Advanced and/or Metastatic Ewing Family of Tumors (EFT) Progressing After at Least One Prior Line of Chemotherapy	Completed	No Results Available	Ewing’s Sarcoma, Primitive Neuroectodermal Tumor (PNET), Askin’s Tumor of the Chest Wall, Extraosseous Ewing’s Sarcoma (EOE)	Drug: Zalypsis	Phase 2	17	Interventional
NCT00588510	Detection of Circulating Osteosarcoma Tumor Cells in the Blood of Patients Using the Polymerase Chain Reaction	Completed	No Results Available	Osteosarcoma	Blood draw		59	Observational
NCT04512495	Circulating “Cancer Cells/Macrophage” Hybrid Cells in Patients With Sarcoma?	Recruiting	No Results Available	Sarcoma	Blood draw	Not Applicable	60	Interventional
NCT03570437	Does Cediranib With Paclitaxel, or Cediranib and Olaparib, Treat Advanced Endometrial Cancer Better Than Paclitaxel?	Recruiting	No Results Available	Carcinosarcoma, Endometrial Neoplasms	Drug: Paclitaxel, Cediranib, Olaparib	Phase 2	129	Interventional
NCT01804634	Reduced Intensity Haploidentical BMT for High-Risk Solid Tumors	Recruiting	No Results Available	Refractory and/or Relapsed Metastatic Solid Tumors	Drug: Cyclophosphamide, Fludarabine, Melphalan, Tacrolimus Low dose total body irradiation	Phase 2	60	Interventional
NCT04214457	Development of a Predictive Model for Early Differential Diagnosis of Uterine Leiomyomas and Leiomyosarcomas	Recruiting	No Results Available	Leiomyoma, Leiomyosarcoma	Biopsy and peripheral blood collection		1000	Observational
NCT00898781	Study of Circulating Cancer Cells in Patients with Metastatic Breast, Ovarian, Colon, or Pancreatic Cancer	Terminated	No Results Available	Breast Cancer, Colorectal Cancer, Ovarian Cancer, Pancreatic Cancer			80	Observational

Objective Remission Rate (ORR); Progression Free Survival (PFS); Overall Survival (OS); Duration of Response (DOR); Disease Control Rate (DCR); Complete Response (CR), Partial Response (PR), Event-Free Survival (EFS), Maximum Observed Plasma Concentration (Cmax), Treatment-emergent Adverse Events (Aes), Serious Adverse Events (SAEs), Time to Reach Maximum Observed Plasma Concentration (Tmax), Plasma Decay Half-Life (t1/2), Time to Reach Last Quantifiable Concentration (Tlast), Systemic Clearance (CL), Concentration at End of Infusion (Cendinf), Volume of Distribution (Vz), Volume of Distribution at Steady State (Vss), Area Under the Curve (AUC), Insulin-like Growth Factor 1 Receptor (IGF-1R), National Cancer Institute Common Terminology Criteria for Adverse Events, version 5.0 (CTCAE), Maximum Tolerated Dose (MTD), Recommended phase II dose (RP2D), Dose Limiting Toxicities (DLT), Common toxicity criteria from the NCI v4.0 (NCI-CTC, Objective Response (ORR), Clinical Benefit (CBR).

## Data Availability

Not appliacable.
